# Effects of Dietary Protein Level on the Microbial Composition and Metabolomic Profile in Postweaning Piglets

**DOI:** 10.1155/2022/3355687

**Published:** 2022-03-30

**Authors:** Jing Gao, ZeMin Liu, ChenYu Wang, Li Ma, Yongzhong Chen, TieJun Li

**Affiliations:** ^1^Key Laboratory of Agro-Ecological Processes in Subtropical Region, Institute of Subtropical Agriculture, Hunan Provincial Key Laboratory of Animal Nutritional Physiology and Metabolic Process, Chinese Academy of Sciences, Changsha, Hunan, China; ^2^University of Chinese Academy of Sciences, Beijing 100039, China; ^3^Research Institute of Oil Tea Camellia, Hunan Academy of Forestry, Shao shan South Road, No. 658, Changsha 410004, China; ^4^National Engineering Research Center for Oil Tea Camellia, Changsha 410004, China

## Abstract

Since the human and porcine digestive systems have similar anatomical structures and physiological functions, pigs are a useful animal model for studying human digestive diseases. By investigating intestinal metabolites in piglets after weaning, this study attempted to identify the inherent connection between dietary protein levels and changes in the intestinal microbiota of piglets. Casein was employed as the only source of protein for the piglets in this study to avoid the influence of other protein sources. 14 weaning at 28-day-old piglets (6.9 ± 0.19 kg) formed into two dietary groups: 17% casein fed group (LP) and 30% casein fed group (HP). Piglets were allowed to free food and water during the 2-week experiment. Throughout the trial, the piglets' diarrhea index (1: no diarrhea and 3: watery diarrhea) and food intake were noted during the experiment. We discovered piglets fed a high-protein diet developed diarrhea throughout the duration of the research, whereas piglets fed a normal protein diet did not. In addition, the HP group had lower feed intake and body weight than the control group (*P* < 0.05). The HP diet influenced the content of short-chain and branched-chain fatty acids in the colon, including acetate and isovaleric acid. The ileal microbiota's 16S rRNA gene was sequenced, and it was discovered that the relative abundance of gastrointestinal bacteria differed between the HP and control groups. Dietary protein levels influenced bile acid biosynthesis, alpha-linolenic acid metabolism, phospholipid biosynthesis, arachidonic acid metabolism, fatty acid biosynthesis, retinol metabolism, arginine and proline metabolism, pyrimidine metabolism, tryptophan metabolism, and glycine and serine metabolism, according to gas chromatography-mass spectrometry analysis. Furthermore, a correlation analysis of the pooled information revealed a possible link between intestinal metabolites and specific bacteria species. These findings demonstrate that weaned piglets' microbiota composition and metabolites are modified by a high-protein diet and thus inducing severe postweaning diarrhea and inhibiting growth performance. However, the potential molecular mechanism of this regulation in the growth of piglets remains unclear.

## 1. Introduction

Dietary protein is required for animal growth and physiological functioning. In China, the environmental pollution caused by nitrogen (N) excretion and the shortage of protein resources is two critical bottlenecks in the development of the pig industry [1, 2]. Reducing N excretion is attracting many interests from agriculturists. According to National Research Council 2012, 18% crude protein concentration is ideal for growing pigs (22-50 kg). A diet rich in essential amino acids and low in protein improves growth performance and conserves protein sources and lowers nitrogen excretion [3]. Protein restriction possesses several advantages: (1) regulates nutritional signaling pathways in humans and animals to avoid illnesses and extend life span [2], (2) lower-protein diets ameliorates the relationship between protein fermentation and the occurrence of postweaning diarrhea in weaning piglets [4], and (3) changes the relative abundance of the gastrointestinal microbiota of adult piglets to improve their function of intestinal barrier [5]. As a result, the practice of feeding animals a low-protein diet has gained widespread acceptance [6]. However, the modulation of protein in diet concentration on the composition of the gastrointestinal tract (GIT) and metabolomic profile stays unclear.

For decades, mounts of microbiota are colonized in animals' GIT, and GIT microbiota was reported to affect nutrient absorption and GIT health, including carbohydrates, proteins, and fats. Dietary protein, especially diets containing a high concentration of protein, fermented by gut microbiota produces some harmful metabolites, such as amine produced by clostridia, and Bacteroides, hydrogen sulfide produced by *Escherichia coli*, *Enterobacter aerogenes*, and *Proteus vulgaris* [7]. Therefore, results in a significant increase in GIT diseases of humans and other animals [8]. The abounding productions originate from dietary protein fermentation act as substrates for the intestinal microbe, therefore influencing the GIT normal metabolisms. For example, proteins could ferment to SCFAs and BCFAs in the colon, to provide additional energy and regulate the metabolism. On the other side, excessive protein fermentation results in superfluous nitrogen influx and reduces the utilizing efficiency of protein. Thus, choosing a feed ingredient containing suit level of protein could avoid the superfluous indigested protein flowing to the GIT, therefore serving as a food source for bacteria that ferment proteins [9, 10].

In the trial, we hypothesized that the high-protein level diet can influence the microbial composition and the fermentation metabolites of weaned piglets. To address these hypotheses, we conducted an analysis of piglets' intestine content microbiota by combining 16S rRNA technology with metabolomics.

## 2. Materials and Methods

We experimented with Chinese animal welfare guidelines. With the ethical approval code ISA2017030523, the Chinese Academy of Sciences' Animal Care and Use Committee accepted the experimental protocol.

### 2.1. Animal Models

Piglets (Duroc Landrace Large White, 6.9 ± 0.19 kg, 28 d) after weaning were randomly separated into two groups, each with seven repetitions, and all piglets were assigned to individual metabolism bar.

The diets we mixed in this experiment were described in our previous study [11]. The diets were free of antibiotics and growth enhancers, and they met or surpassed NRC standards for all essential amino acids.

Piglets were housed in temperature-controlled incubators for two weeks during the experiment. All animals were given unlimited feeding and drinking water. Before and after the experiment, we recorded the weight of piglets. Besides, feed intake was noted every day for calculating the growth performance index.

### 2.2. Sampling

The piglets were fasted for 24 hours before being killed. Fresh ileum content and fresh colon content were gathered from every piglet into sterile 50 ml centrifuge tubes, and immediately before extraction of total genomic DNA, the samples are subpackaged and then kept at -80°C.

### 2.3. Determine Short-Chain Fatty Acid Content

About 1 g of colon content sample was accurately weighed on a one-tenth balance, and 1.0 mL of ultrapure water was added, vortexed for 45 s, and centrifuged at 16,000 r/min, 18 min. We collected the supernatant and added 25% metaphosphoric acid under a ratio of v : v =9 : 1 for 2, 3, 4, and 5 h until tested. Capillary column gas chromatography was used to determine the amount of SCFA in the colon (GC-14B, Shimadzu, Japan).

### 2.4. DNA Isolation, 16S rRNA Sequence

A QIAamp DNA Stool DNA Minikit was used to test total microbial genomic DNA from ileum content (51504; Qiagen, Hilden, Germany). Applying diluted microbial genomic DNA, Routine PCR was conducted to amplify the V3-V4 region of 16S rDNA [12, 13]. And retrieved PCR products were utilized to build libraries. The libraries were then quantified using Qubit and Q-PCR before being sequenced on a HiSeq2500 PE250. Uparse (v7.0.1001) was used to cluster operational taxonomic units (OTUs). Alpha and beta diversity indices were determined by the Qiime software, which included observed species, Shannon, and Chao1 indices and weighted and unweighted UniFrac distances. The hierarchical grouping of samples was completed using UPGMA. Principal component analysis (PCA) was done with R 2.15.3. A two-sided Student's *t*-test was used to determine the significance of differences in alpha and beta diversity across groups.

### 2.5. Bioinformatics Analysis

The ends of the fragments were derivatized, as well as ligation adapters were connected for both ends, known as the sequence alignment identifier. All high-throughput sequencing reads are processed using barcodes and primer sequences, the resultant reads are screened, and quality filtered further. The abundance-based coverage estimator (ACE), Shannon, the bias-corrected (Chao) richness estimator, and Simpson diversity indices were determined with the Mothur program (http://www.mothur.org) [14].

### 2.6. Metabolomics Analysis

Individually powdered tissues (100 mg) were homogenized with liquid nitrogen and resuspended in prechilled 80% methanol, after centrifuging at 16,000 g, 4°C for 25 minutes, and then transferred on ice for a while. LC-MS grade water was used to dilute the supernatants to a final concentration of 53% methanol. UHPLC-MS/MS analysis was completed by Novogene Co., Ltd. (Beijing, China).

After dehybridization analysis of the original data, fragment ions, molecular ion peaks, additive fragment ions, and the normalized data were utilized to infer the molecular formula. The statistical program R (version R-3.4.3) was used to conduct the analysis.

The KEGG database (https://www.genome.jp/kegg/pathway.html) was used to annotate these metabolites, and principal component analysis (PCA) and partial least squares discriminant analysis (PLS-DA) were utilized at metaX. Ggplot2 in R was used to select metabolites of interest using volcano plots based on log2 (fold change) and -log10 (*P* value) of metabolites. Differential metabolites were identified as those with VIP > 1 and a *P* value of <0.05, as well as fold change2 or FC0.5.

### 2.7. Statistical Analysis

Data were statistically analyzed by SPSS 23.0 in a randomized block group design, with diet as the only factor. To compare different parameters across the two treatments, an unpaired, two-tailed Student's *t*-test was utilized. At *P* < 0.05, the data analysis was declared statistically significant [15].

## 3. Results

### 3.1. Growth Performance

**Figure 1:**
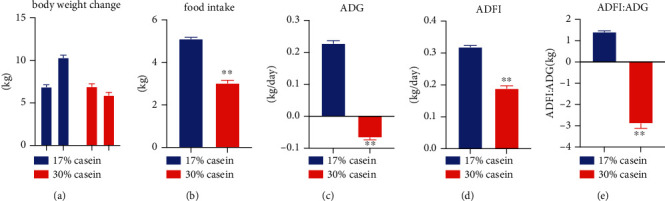
High casein concentration reduced the growth performance of weaned piglets. (a) Body weight change. (b) Food intake. (c) ADG. (d) ADFI. (e) ADFI/ADG. Data are expressed as the mean ± SEM, *n* = 7.

According to the result, we found persistent diarrhea in the HP piglets but no diarrhea in the control group during the whole trial (*P* < 0.05) (Table 1). The result indicated that high-protein concentration feed notably restrained the body weight (BW, kg) (*P* < 0.05), food intake (FI, kg) (*P* < 0.01), average daily feed intake (ADFI, kg/day) (*P* < 0.01), average daily gain (ADG, kg/day) (*P* < 0.01), and feed efficiency (G : F ratio, kg) (*P* < 0.01) compared with the control group (Figures 1(a)–1(e)).

### 3.2. Short-Chain Fatty Acid Analysis

**Figure 2:**
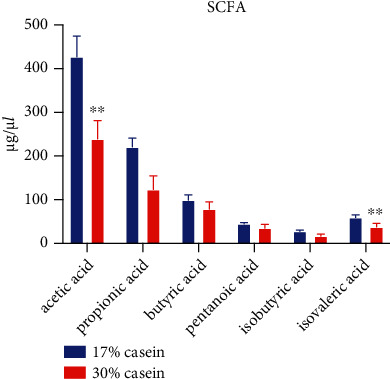
High casein concentration decreased the concentration of SCFA in weaned piglets. Colon SCFA relative abundance (mmol/l) in postweaning piglets (*n* = 7). Data are shown as the mean ± SEM, *n* = 7.

The concentrations of short-chain fatty acids (SCFAs) and branch-chain fatty acids (BCFAs), including acetic acid and isovalerate in the colon, was significantly decreased in the high dietary protein level diet group piglets (Figure 2).

### 3.3. Intestinal Microbiota

**Figure 3:**
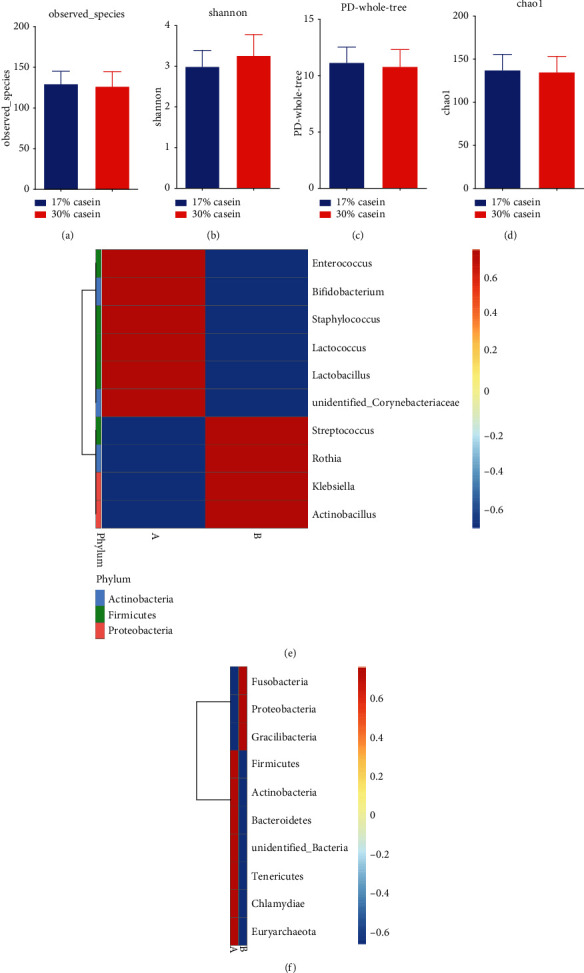
High casein concentration influenced gut microbial diversity of piglets. (a) Observed species; (b) phylogenetic diversity (PD); (c) Shannon H index; (d) Chao1 index. (a) represents the LP group; (b) represents the HP diet group. (e) Genus and (f) phylum in the colon of piglets (*n* = 7).

Each colon sample yielded an average of 6878.80 ± 2117 sequences. We retrieved a typical of 690 ± 100 OTUs per piglets using pairwise identity limits of 97%. Differences in observed species Chao1, ACE, Simpson, Shannon, and good-coverage that represents alpha diversity were calculated. However, the high-casein diet appeared to inhibit ileal microbiota diversity compared to control piglets, but the difference showed insignificant (PD, 11.16 ± 1.39 vs. 10.81 ± 1.51, *P* = 0.8; H, 2.99 ± 0.4 vs. 3.26 ± 0.57, *P* = 0.6; and Chao1, 137.32 ± 18.17 vs. 134.94 ± 18.3, *P* = 0.9) (Figures 3(a)–3(d)). To confirm differences in *β*-diversity of microbiome groups, we performed principal component analysis (PCoA) to obtain weighted and unweighted UniFrac distance matrices produced for the sample set, while the results of weighted (*P* = 0.8) and unweighted UniFrac distances (*P* = 0.17) implied that no significant microbial community structure difference existed between the two treatments (data not shown).

The relative abundance of GIT microbial between different treatments produced differences on phylum and genus levels. In each data set, the three largest phyla represented *Firmicutes* (*P* > 0.05), *Proteobacteria* (*P* > 0.05), and *Actinobacteria* (*P* > 0.05); however, no significant differences were found after *t*-test correction (*P* > 0.05). Besides, the concentration of the top three genera, *Lactobacillus* (*P* > 0.05), *Streptococcus* (*P* < 0.05), and *Lactococcus* (*P* > 0.05), was at the same level in two datasets (*P* > 0.05). Somehow, diet changed into HP level reduced the content of *Lactobacillus* and *Bifidobacterium* (*P* > 0.05) and enhanced the level of pathogen bacteria, such as *Fusobacteria* and *Proteobacteria* (*P* > 0.05) (Figures 3(e) and 3(f)).

### 3.4. Metabolomic Profiles

**Figure 4:**
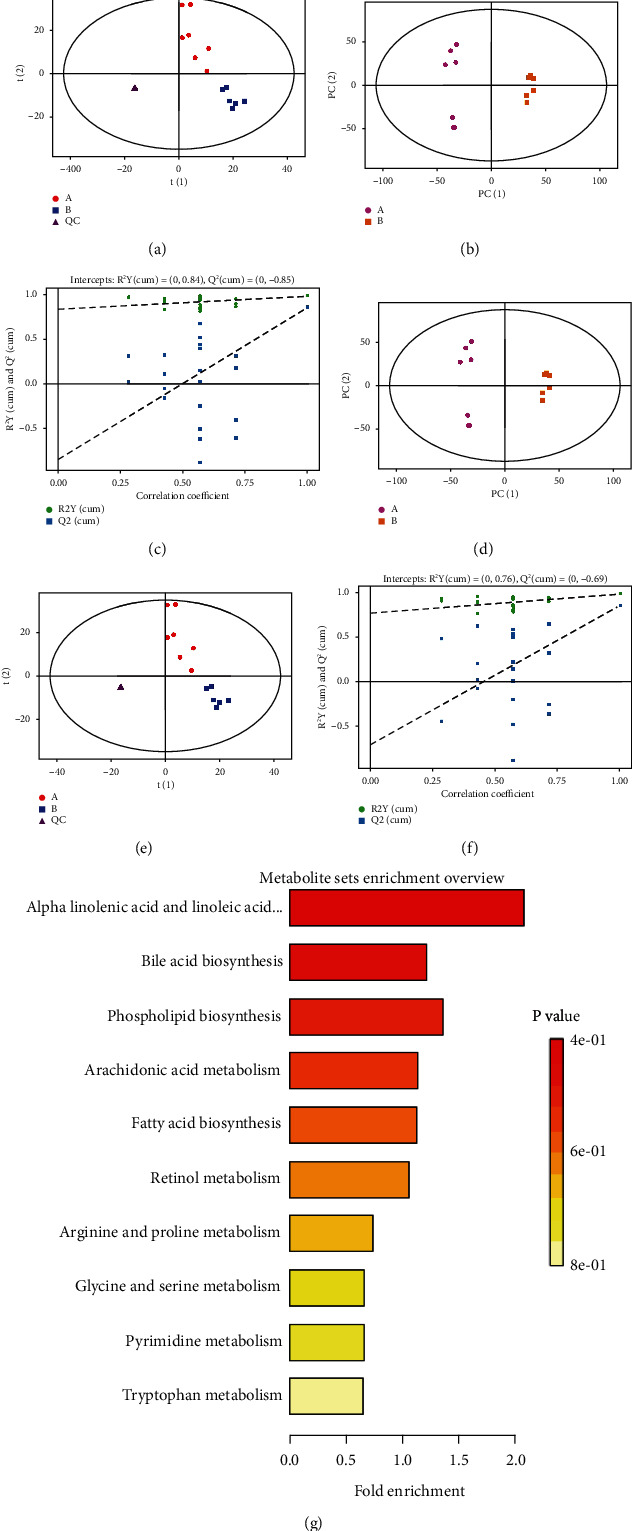
High casein concentration influenced biomarker metabolites in piglets. PLS-DA and OPLS-DA of microbial metabolites in colonic contents from piglets fed LP and HP. The (a) PLS-DA score plot is in negative ion mode. The (b) OPLS-DA score plot is in negative ion mode. The (c) PLS-DA score plot is in negative ion mode, and (d) the OPLS-DA score plot is in positive ion mode in different protein treatments. Validation plot of OPLS-DA model in (e) negative ion model and in (f) positive ion mode in the HP protein group. (g) Analysis of metabolic pathway enrichment. An overview of the metabolites that were shown to be abundant in piglets fed the HP diet compared to the LP diet.

We conducted the unsupervised analysis (PCA) of colon content in the two groups. The quality control samples (QCs) reflect the controlled stability of the experimental sample assay and instrumentation, clustered together and separated from the experimental colon content as shown in the figure. We found that R2 (cum) was 0.91, and Q2 (cum) was -0.42 in the negative ion model while 0.89 and -0.51 in the positive ion model, respectively, demonstrating the validity of both models and that the colonic contents of the LP and HP groups were completely separated. To prevent the overfitting of the data, a reciprocity process was conducted (Figures 4(a)–4(f)).

The importance of the combined variables for prediction (VIP) value > 1, and the concentration (intensity) in two treatments showed markedly different (*P* < 0.05). Differentially expressed metabolites were identified as multiple metabolites. There were 64 metabolites identified as putative important regulators, with 1-45 coming from the negative ion mode database, while 46-64 from the positive mode database. Selected potential biomarkers in two groups were gone through a heatmap work. At this point, the LP group and HP group piglets could be completely separated. Compared to the LP group, piglets in the HP group expressed higher concentration of the following metabolites, (2-oxo-2,3-dihydro-1H-indol-3-yl)acetic acid, cucurbitacin I, L--hydroxyisovaleric acid, orotate, pentanoate, pentanoic acid, 2-methyl-4,5-benzoxazole, 2-methyl-4,5-benzoxazole, 5-ethyl-5-(pentan-2-yl)-1-((2S,3R,4S,5S,6R)-3,4,5-trihydroxy-6-(hydroxymethyl)tetrahydro-2H-pyran-2-yl)pyrimidine 2,4,6(1H,3H,5H)-trione, damascenine, Trp-Pro-Ser, Ala-Ala-Glu, 3,6-dimethoxyestra-1,3,5(10),6,8-pentaene-17 beta-carboxylic acid methyl ester, eremophilenolide, D-erythro-Sphingosine C-20, PE(16 : 0/0 : 0), LysoPC(14 : 0), lucanthone, N-methyl-1H-indole-3-propanamide, methyldopa, 3-hydroxy-5-chola-8(14), 11-dien-24-oic acid, oseltamivir, retinol, 2-(4-methyl-5-thiazolyl)ethyl decanoate, mupirocin, hericenone H, cadiamine, leiokinine A, aklomide, Met-Gly-Pro-Thr, 2(N)-methyl-norsalsolinol, arachidonoylmorpholine, and 5-androstan-3-ol-17-one sulfate, whereas piglets fed HP diet showed lower concentration of the following metabolites, Ile Pro, 19-norandrosterone, 13-+A12+A33+A33:A55, 17-U-46619, 9-HODE, 9-HOTrE, azelaic acid, beta-hydroxymyristic acid, glycocholate, N-Ac-Tyr-Val-Ala-Asp-CHO, sebacic acid, taurocholic acid, creatine, goniothalesdiol, 14,15-LTA4, tephrowatsin B, Dimefuron, PGA1, 3-(2-methylpropanoyloxy)-8-(3-methylbutanoyloxy)-9,10-epoxy-p-mentha-1,3,5-triene, prostaglandin I2, 3-indoleacetic acid, 5-propylideneisolongifolane, 11-dehydro-TXB3, Kamahine C, 3-(2,4-cyclopentadien-1-ylidene)-5alpha-androstan-17beta-ol,(9R,13R)-12-oxophytodienoic acid, stearidonic acid, 5′-methoxyhydnocarpin-D, Trp-Glu-Glu, Asn-Arg-Lys-Ala, and 6-[3]-ladderane-1-hexanol (for details, see Supplementary Document 1, The differences-metabolites between two groups).

To achieve common pathway information based on an obtained potential biomarker, KEGG analysis was conducted. As we described before, the *P* value was positively correlated with discriminating metabolites, while the impact value of topological analysis was positively correlated with discriminating metabolites. According to our results, when piglets gave HP diet two weeks, the metabolic pathway of bile acid biosynthesis, alpha-Linolenic acid metabolism, phospholipid biosynthesis, arachidonic acid metabolism, fatty acid biosynthesis, retinol metabolism, arginine and proline metabolism, pyrimidine metabolism, tryptophan metabolism, and glycine and serine metabolism were influenced (Figure 4(g)).

### 3.5. The Ileum Microorganism and the Intestinal Metabolome Correlation Analysis

**Figure 5:**
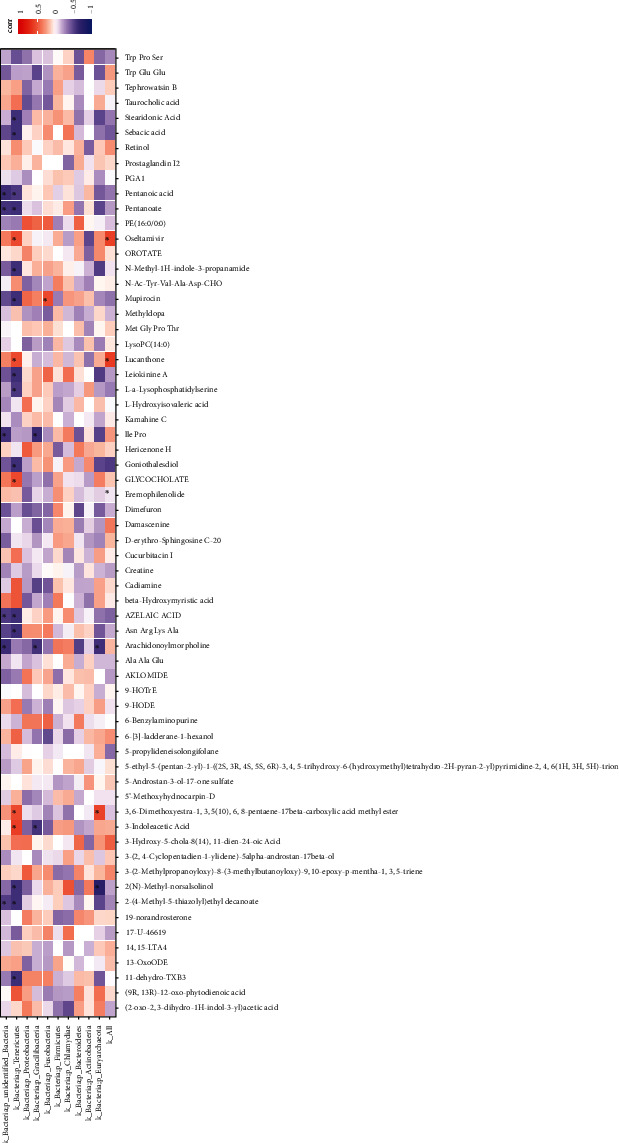
The ileum microorganism and the intestinal metabolome correlation analysis in piglets. The color scheme is based on the distribution of Pearson correlation coefficients.

To clarify the relevance between ileum microbiota (phylum level) and metabolites (VIP > 1.0, *P* < 0.1), correlations between taxa affected by different protein level treatments were calculated by Pearson's correlation coefficient. The result showed that pentanoate was found to have a negative relationship with *Firmicutes*, *Bacteroidetes*, and *Actinobacteria*, while having a positive relationship with *Proteobacteria*, *Fusobacteria*, and *Gracilibacteria*. Azelaic acid, sebacic acid, stearidonic acid, and glycocholate were positively associated with *Firmicutes*, *Bacteroidetes*, and *Actinobacteria* and negatively associated with *Fusobacteria* and *Gracilibacteria*. Ile-Pro-and Asn-Arg-Lys-Ala were negatively associated with *Proteobacteria*, *Fusobacteria*, and *Gr*acilibacteria. L-a-Lysophosphatidylserine was adversely connected to *Firmicutes* and *Bacteroidetes* but positively connected to *Proteobacteria*, *Fusobacteria*, *Gracilibacteria*, and *Actinobacteria*. 3-indoleacetic acid and N-methyl-1H-indole-3-propanamide were negatively associated with *Actinobacteria*, *Proteobacteria*, *Bacteroidetes*, *Fusobacteria*, and *Gracilibacteria* but were positively associated with *Firmicutes* (Figure 5).

## 4. Discussion

In recent researches, based on the current swine industry practices, the environmental pollution caused by nitrogen (N) excretion and the shortage of protein resources are two critical bottlenecks in the development of the pig industry [16, 17]. A low-protein feed was widely conducted to test the relationship between the gastrointestinal microbiota community and metabolomic profiles of the pigs [18, 19]. According to our results, a high-protein feed notably decreased the growth performance of the piglets after weaning. However, the diversity of the ileum microbiota was not altered by the supplement of the protein concentration in the diet, although the abundance of several genera was influenced. Moreover, the metabolites of the colon were modulated by the different protein levels in the two diets.

Multiple studies indicated that dietary protein levels in diet influence the composition and abundance of gastrointestinal microbiota and their fermented metabolites, thus modulating the host health [20, 21]. In our study, the different protein level diets only altered several genera abundance. However, it had no effect on the ileum's gut microbial diversity. *Firmicutes* and *Bacteroidetes* phyla and *Actinobacteria* phylum were an essential component of the gut microbiome in human gut microbes [22]. According to previous reports, *Firmicutes*, *Bacteroidetes*, and *Actinobacteria* bacterial group in the GIT play act as a key factor in the nutritional absorption [23, 24]. *Actinobacteria* are Gram-positive filamentous bacteria. *Actinobacteria* are indicated to act as potential probiotics and are important in the maintenance of gut homeostasis [25, 26]. Dozens of studies showed the *Fusobacterium* genus as a pathogen bacterium; its abundance is strongly associated with colorectal cancer [27, 28], while *Proteobacteria* are a type of Gram-negative bacteria that belong to the phylum Proteobacteria. Early reports have shown that *Proteobacteria* were an abundant factor in human diseases [29]. *Proteobacteria* include a series of species, such as the genera *Neisseria*, *Yersinia*, *Agrobacterium*, and *Escherichia.* Among these bacteria, Escherichia coli is indicated very closely associated with diarrhea in humans and pigs [30, 31].

Members of the genus *Fusobacterium* consist of the *Fusobacteria* phylum and belong to Gram-negative anaerobes [32]. Previous studies indicated that *Fusobacteria is* closely associated with intestine diseases [33]. *Fusobacterium* acts as effective damaging causes that induce intestinal injury in piglets [34, 35]. In our study, an HP diet reduced the content of *Firmicutes*, *Bacteroidetes*, and *Actinobacteria* at the phylum but improved the concentration of *Fusobacteria* and *Proteobacteria* at the phylum level. The increased relative abundance of *Fusobacterium*, *Gracilibacteria*, and *Proteobacteria* in the ileum content may notably enhance postweaning diarrhea of piglets, which is possibly associated with the redundant indigested protein in the intestine.

Mammals and other animals' *Bifidobacteria* and *Lactococcus/Lactobacillus* (and some strains) of the gastrointestinal microbiota are found to act as health-promoting factors [36]. The genus *Bifidobacteria* belongs to the *Actinobacteria* phylum, while the genus of *Lactococcus*/*Lactobacillus* belongs to *Firmicutes* phylum are the two most abundance microbiota in the intestine, especially in the newborns GIT [37]. They have reported contributions to the development of the host's intestinal physiology, such as maturation of the immune system, digestive system, bacterial colonization, and pathogen exclusion [38–42]. The digestion of many nutrition intakes by the host was based on the activity of *Bifidobacteria* and *Lactobacillus.* Many kinds of research showed that many species of *Lactobacillus* made a contribution to the regulation of cell layer integrity and suppressed intestine injure caused by Salmonella LPS administration through influencing the activity of tight junction protein 1 (ZO-1) [43]. Another important function of *Bifidobacteria* and *Lactobacillus* is to maintain the pH of the gastrointestine [44]. In this experiment, the concentration of *Bifidobacteria*, *Lactococcus*, and *Lactobacillus* genera was decreased in HP diet-treated piglets. The genera of *Bifidobacteria* and *Lactobacillus* belong to lactic acid bacteria. Previous studies demonstrated the beneficial effects of lactic acid bacteria, including manipulation of the intestinal microecology, prevention of the colonization of GIT pathogen bacteria, strengthening the intestinal mucosal immunity, and sustaining intestinal barrier function [38, 45–47]. Consist with the existed researches, our study also indicated that the protein level of diet was closely connected with the GIT microbiome composition. That is to say, more protein mammal intake, less lactic acid bacteria species mammal's intestine has. However, the relative abundance of *Streptococcus*, *Klebsiella*, and *Actinobacillus* genera was increased in the ileum. Genus *Streptococcus* belongs to lactic acid bacteria, and some species represent the same beneficial effect with *Lactobacillus*, but others in the treatment are reported as potential pathogens [48, 49]. Meanwhile, many species of genus *Klebsiella* are famous for most relevant opportunistic pathogens [50]. The exact relationship between dietary protein level and genera *Streptococcus* and *Klebsiella* should be evaluated in future studies. Therefore, *Fusobacteria* and *Proteobacteria* phyla and *Klebsiella* genus occupy a dominant phylum and genus status in HP diet piglets and improve affix and invade ability of intestinal cells, thus improving the regulation effect and the metabolic pathways of the GIT. However, this presupposition has not been further proved. Give postweaning piglets high-protein level treatment, provide excessive undigested dietary protein to piglets' intestinal microbiota, and strongly promote the development of intestinal diseases, including postweaning diarrhea.

Indigestive proteins that arrived in the colon are fermented to BCFAs such as isobutyrate and isovalerate. Besides, AAs could also ferment to SCFAs, mainly acetate and propionate production [51, 52]. SCFAs could take part in many organisms' metabolic pathways act as an important factor to manage metabolic disorders and the immunity system, including GIT, endocrine, and nervous [53]. Although SCFAs are not regarded as a major energy source for human and animals, previous reports have shown SCFA production significantly regulates the host energy metabolism [54, 55]. However, redundant fermentation of proteins in the colon has indicated induced intestine diseases, such as diarrhea and colon cancer. Acetate is demonstrated to participate in the regulation of adipose tissue deposition [56]. Combined with the analysis of the intestine microbiota, we found the concentration of *Bifidobacteria*, *Lactococcus*, and *Lactobacillus* genera were decreased in HP diet-treated piglets. The genera of *Bifidobacteria* and *Lactobacillus* belong to lactic acid bacteria, while the most abundant production of lactic acid bacteria is acetic acid. Isovalerate was closely associated with increased ammonia levels and accumulated toxic metabolites [57]. Consist with previous reports, the results indicated that an HP diet reduced the concentration of acetic acid. However, decreased isovalerate concentration compared to LP diet piglets is probably because of the content of digested protein.

According to the results of metabolic pathway analysis, bile acid metabolism, arachidonic acid metabolism, alpha-linolenic acid metabolism, fatty acid biosynthesis, tryptophan metabolism, arginine and proline metabolism, and Gly and the Ser metabolism were regarded as the pivotal pathways that distinguished between the two treatments. In the HP group piglets, fatty acid biosynthesis differed from this pathway in the LP group, which could be proved by the increased concentration of pentanoate and pentanoic acid. Isovalerate is a short-chain fatty acid generated by members of the gut microbiota through amino acid fermentation. Protein could ferment to 5-amino valerate through hydrolysis under the help of intestinal microbiota, and proline could also break down to 5-amino valerate. 5-Amino valerate then fermented to pentanoate via intestine bacteria fermentation [57, 58]. As mentioned before, indigested protein and AA could be fermented by gut bacteria to SCFAs and BCFAs. In this study, acetic acid and isovalerate were markedly reduced in the high dietary protein feed piglets' colon. Isovalerate was closely associated with increased ammonia levels and accumulated toxic metabolites. Maybe the difference between fatty acid metabolism in the two treatments was closely associated with redundant ammonia from the amount of protein indigested by intestinal microbiota in the HP group. In the HP group piglets, the metabolism of alpha-linolenic acid differed from this pathway in LP piglets, which could be proved by the decreased concentration of azelaic acid, sebacic acid, and stearidonic acid. Alpha-linolenic acid is a product of *Bifidobacteria* and is involved in the anti-inflammatory process [59, 60]. Correction of gut microbiota and metabolome results indicated that *Bifidobacteria* and *Lactobacillus* with their products were decreased, and the metabolome results were consistent with the microbe results.

In the HP group piglets, bile acid biosynthesis and arachidonic acid metabolism differed from this pathway in the LP group, which could be proved by the decreased concentration of glycocholate and arachidonoylmorpholine. Bile acids were indicated to participate in animals' digestive process, regulated the gut microbiota's composition and anti-inflammatory properties [41, 61]. Previous researches reported that unbalanced bile acid biosynthesis was closely associated with liver diseases [60]. In our study, decreased concentration of glycocholate indicated unregulated bile acid biosynthesis. And this may be relative to the amounts of accumulated toxic metabolites in the colon, which affected the liver function of the piglets in the HP group. According to previous researches, arachidonoylmorpholine acts as an important role that contributes to the function of human tissues, immune system, and digestive process, especially in infant period [62]. Arachidonoylmorpholine is a member of bile acid. And arachidonic acid metabolism was reported involved in kidney inflammation and arachidonic acid decomposition to prostaglandins, thromboxane, and leukotrienes in the kidney. However, the increased concentration of prostaglandins, thromboxane, and leukotrienes could trigger kidney inflammation, thus inducing kidney injury [63]. In the study, the HP group piglets exhibited sustained diarrhea during the whole experiment, and there was no diarrhea happened among LP group piglets. One outstanding characteristic of diarrhea is impaired renal function. The increased concentration of arachidonoylmorpholine may induce disordered arachidonic acid metabolism and aggravate the extent of postweaning diarrhea by a high-protein diet.

In the HP diet piglets, the arginine, proline metabolism, tryptophan metabolism, and glycine and serine metabolism were influenced, which informed from the decrease of Ile-Pro, Asn-Arg-Lys-Ala, and 3-indoleacetic acid. Tryptophan could provide ingredients for several bioactive compounds, including melatonin, serotonin, kynurenine, and picolinic acid [64]. And tryptophan is involved in the physiological function in the GIT, regulating intestinal permeability and secretion [65, 66]. A previous study showed tryptophan could alleviate colitis [67]. Moreover, tryptophan was engaged in microbiota diversity in the GIT of pigs [68]. Arginine improves the proliferation of intestinal epithelial cells and gives birth to a successful immune response, thus decreasing intestinal injury [69, 70]. Also, arginine is a senor of mammalian target of rapamycin (mTOR) [71]. mTOR signaling pathway is involved in many animal diseases, such as autophagy. And mTOR signaling pathway is closely associated with nutrients [72, 73]. Amino acid, such as Arg, Ser, and Ile, the essential components for protein synthesis and products of protein ferment, could regulate the mTOR signaling pathway [74]. Lysine is another essential component for protein synthesis in humans and animals. An early study indicated decreased concentration of lysine inhibited feed intake and intestinal absorption and metabolism of amino acids in piglets. Lysine concentration is positive with the levels of the phyla *Actinobacteria*, which consist with our results. Proline was reported to contribute to cytodifferentiation and synthesis of some amino acids in the gut of postweaning piglets [75–77]. Supplementation of proline could increase immune function, growth performance, gastrointestinal tract digestibility, and ammonia detoxification in postweaning piglets [75, 78]. Thus, amino acids, especially proline, lysine, serine, arginine, and glycine, participate and modulate the GIT microecology and host immune process to provide protective effects on human and animal health.

## 5. Conclusion

With all that results, we explored the regulation of a high protein concentration diet on the GIT composition and metabolite in postweaning piglets. It is implied an HP diet significantly decreased the growth performance of postweaning piglets and further affected the concentration of SCFAs, BCFAs, microbial composition, and metabolite profiles. Together, these above influences might be associated with the degree of digestive protein; redundant indigested protein modulated the composition of the GIT, thus altering the metabolite profiles. These changed GIT microbiota and colon metabolites may be used as a potential target for nutrition regulations. We confirmed that the regulation of an LP diet on the growth of weaned piglets should be further questioned.

## Figures and Tables

**Table 1:** The diarrhea rate of two group piglets.

Index	17% casein	30% casein	SEM	*P* value
Diarrhea rate	0.23	0.806	0.1146	<0.01

## Data Availability

The data used to support the findings of this study are available from the corresponding author upon request.

## Abbreviations

SCFAs: Short-chain fatty acids
BCFAs: Branch-chain fatty acids
GC-MS: The gas chromatography-mass spectrometry
N: Nitrogen
GIT: Gastrointestinal tract
AA: Amino acid
G : F: Average daily gain : average daily food intake
PCA: Principal component analysis
OPLS-DA: Orthogonal partial least sequences discriminant analysis
OTU: Operational taxonomic units.

## Supplementary Materials

Supplementary Materials Supplementary Document 1: the differences-metabolites between two groups.
