# Supplementary material for: Effects of Dietary Protein Level on the Microbial Composition and Metabolomic Profile in Postweaning Piglets
Source: Oxid Med Cell Longev. 2022 Mar 30;2022:3355687. doi: 10.1155/2022/3355687 (PMC8986435; doi:10.1155/2022/3355687)
Supplement: Supplementary Materials — Supplementary Document 1: the differences-metabolites between two groups. [file 3355687.f1.doc]

Supplementary Document 1, The differences-metabolites between two groups

| **name** | **rt** | **mz** | **score** | **VIP** | **P-VALUE** | **Q-VALUE** |
| --- | --- | --- | --- | --- | --- | --- |
| Creatine | 33.794 | 132.0766346 | 0.999322077 | 1.560959172 | 0.018964001 | 0.08093184 |
| Goniothalesdiol | 258.7705 | 267.1197511 | 0.998767692 | 1.35024145 | 0.008445872 | 0.053327594 |
| 14,15-LTA4 | 449.593 | 319.2236723 | 0.998567231 | 2.087131087 | 0.002899773 | 0.029995783 |
| 5-ethyl-5-(pentan-2-yl)-1-((2S,3R,4S,5S,6R)-3,4,5-trihydroxy-6-(hydroxymethyl) tetrahydro-2H-pyran-2-yl) pyrimidine-2,4,6(1H,3H,5H)-trione | 147.6255 | 389.1907442 | 0.998415077 | 2.015536566 | 0.000222059 | 0.008123552 |
| Tephrowatsin B | 408.0235 | 337.1764721 | 0.997904846 | 1.300139271 | 0.028480677 | 0.099803501 |
| Dimefuron | 283.212 | 339.1218879 | 0.997193077 | 2.069097688 | 5.46426E-05 | 0.004542561 |
| Damascenine | 143.85 | 196.09666 | 0.997191615 | 1.671817067 | 0.00191605 | 0.024276843 |
| Trp Pro Ser | 262.995 | 389.1847188 | 0.996612308 | 2.045970294 | 0.000364547 | 0.010049124 |
| Ala Ala Glu | 36.5055 | 290.1339665 | 0.996196462 | 1.660360357 | 0.002707039 | 0.029046886 |
| PGA1 | 376.883 | 337.234055 | 0.995742692 | 1.466906882 | 0.008797147 | 0.054403955 |
| 3-(2-Methylpropanoyloxy)-8-(3-methylbutanoyloxy)-9,10-epoxy-p-mentha-1,3,5-triene | 343.576 | 335.1820541 | 0.994817231 | 2.103736979 | 0.00037826 | 0.010233675 |
| Prostaglandin I2 | 302.778 | 353.2291115 | 0.994662538 | 1.913814887 | 0.009872469 | 0.057540066 |
| 3-Indoleacetic acid | 256.199 | 176.0704214 | 0.991995846 | 1.691146202 | 0.030546678 | 0.1033368 |
| 5-Androstan-3-ol-17-one sulfate | 165.1185 | 371.1916247 | 0.991847154 | 1.881405003 | 0.001127248 | 0.01859527 |
| 3,6-Dimethoxyestra-1,3,5(10),6,8-pentaene-17beta-carboxylic acid methyl ester | 191.375 | 355.186392 | 0.991215385 | 1.374231847 | 0.037446908 | 0.113876397 |
| 5-propylideneisolongifolane | 458.389 | 247.2415296 | 0.990685769 | 1.444493547 | 0.047127849 | 0.127152768 |
| 11-dehydro-TXB3 | 346.785 | 349.1977809 | 0.990273846 | 1.637135738 | 0.036793434 | 0.112950476 |
| Eremophilenolide | 426.889 | 235.1688054 | 0.990023462 | 1.827143065 | 0.002720478 | 0.029115341 |
| D-erythro-Sphingosine C-20 | 393.0685 | 328.3201469 | 0.988487538 | 1.609236041 | 0.030547955 | 0.103338909 |
| PE(16:0/0:0) | 414.529 | 454.2916225 | 0.988135538 | 1.905392369 | 0.002133252 | 0.025750351 |
| LysoPC(14:0) | 377.915 | 468.3071921 | 0.987834538 | 1.510727375 | 0.042002465 | 0.120211741 |
| Lucanthone | 181.7265 | 341.1699335 | 0.987055154 | 1.801116016 | 0.000422864 | 0.01078932 |
| L-a-Lysophosphatidylserine | 394.854 | 524.2984532 | 0.953933538 | 1.785560788 | 0.010263423 | 0.081739577 |
| N-Methyl-1H-indole-3-propanamide | 255.664 | 203.1175692 | 0.985608923 | 1.543884131 | 0.009314157 | 0.055937082 |
| Kamahine C | 214.76 | 269.1281435 | 0.985601231 | 1.078816778 | 0.026606172 | 0.096371007 |
| Methyldopa | 144.396 | 212.0914142 | 0.984775231 | 1.605970424 | 0.001566368 | 0.021784723 |
| 3-Hydroxy-5-chola-8(14),11-dien-24-oic Acid | 246.8465 | 373.2768569 | 0.983192846 | 1.542147094 | 0.007880221 | 0.051496098 |
| Oseltamivir | 187.903 | 313.2024158 | 0.983187846 | 2.093493579 | 4.46542E-05 | 0.004200724 |
| 3-(2,4-Cyclopentadien-1-ylidene)-5alpha-androstan-17beta-ol | 391.787 | 339.2673424 | 0.981662154 | 1.939213886 | 0.006115654 | 0.045092623 |
| Retinol | 404.4805 | 269.2257615 | 0.979835538 | 1.917745063 | 0.017966899 | 0.078870698 |
| (9R,13R)-12-oxo-phytodienoic acid | 424.112 | 293.2103864 | 0.977469923 | 1.601481879 | 0.017954355 | 0.078844047 |
| 2-(4-Methyl-5-thiazolyl)ethyl decanoate | 182.969 | 298.179618 | 0.974051385 | 1.245285108 | 0.004119178 | 0.035918147 |
| Mupirocin | 416.969 | 501.3018552 | 0.973390538 | 1.311974052 | 0.001856757 | 0.023848822 |
| Ile Pro | 169.392 | 229.1543256 | 0.970172308 | 1.889416175 | 0.001130202 | 0.018619473 |
| Hericenone H | 473.4275 | 595.3953713 | 0.969421154 | 1.550026543 | 0.009631288 | 0.056859366 |
| Stearidonic Acid | 400.681 | 277.2156689 | 0.969139077 | 1.838204711 | 0.007095259 | 0.048731448 |
| 5'-Methoxyhydnocarpin-D | 319.726 | 495.1270699 | 0.968996846 | 1.752722003 | 0.004571538 | 0.038271628 |
| Cadiamine | 183.866 | 283.2009306 | 0.965343615 | 1.80257369 | 0.003565851 | 0.033358611 |
| Trp Glu Glu | 263.105 | 463.1827492 | 0.963261385 | 1.984301414 | 0.009547837 | 0.056619722 |
| Met Gly Pro Thr | 372.597 | 405.1797831 | 0.963206154 | 1.298965542 | 0.033275458 | 0.107649258 |
| 2(N)-Methyl-norsalsolinol | 183.866 | 180.1016808 | 0.962303154 | 1.23600181 | 0.000190834 | 0.007626424 |
| 19-norandrosterone | 487.3555 | 277.2158021 | 0.959403 | 1.52875685 | 0.042942516 | 0.121494732 |
| Leiokinine A | 534.211 | 232.1327736 | 0.958402077 | 1.983817994 | 1.80726E-05 | 0.002805823 |
| Asn Arg Lys Ala | 295.964 | 488.2969289 | 0.958280769 | 1.728548417 | 0.025198419 | 0.093637438 |
| 6-[3]-ladderane-1-hexanol | 435.48 | 263.2364316 | 0.955132538 | 1.537403447 | 0.01871804 | 0.080434068 |
| Arachidonoylmorpholine | 487.599 | 374.3020609 | 0.951048 | 1.453775999 | 0.031565458 | 0.104990563 |
| OROTATE | 34.3193 | 155.0096479 | 0.998744154 | 1.03974798 | 0.007904489 | 0.072953147 |
| Pentanoate | 77.4643 | 101.0607364 | 0.997897923 | 1.92284502 | 0.002236695 | 0.043679144 |
| 13-OxoODE | 365.9425 | 293.2119498 | 0.997548615 | 2.142424229 | 0.000639795 | 0.024892311 |
| 9-HODE | 389.726 | 295.2273789 | 0.995307692 | 2.005318884 | 0.006122668 | 0.066058353 |
| GLYCOCHOLATE | 251.704 | 464.3009314 | 0.992748 | 1.935711878 | 0.022953399 | 0.117421817 |
| N-Ac-Tyr-Val-Ala-Asp-CHO | 320.284 | 491.2099358 | 0.991381769 | 1.73568031 | 0.046541012 | 0.161633977 |
| AZELAIC ACID | 73.3564 | 187.097336 | 0.986786615 | 1.503249697 | 0.004175987 | 0.055924416 |
| 9-HOTrE | 346.9205 | 293.2119729 | 0.978304154 | 1.496031788 | 0.039066458 | 0.149386409 |
| Sebacic acid | 175.414 | 201.1130305 | 0.976196692 | 1.687861735 | 0.015911252 | 0.09976179 |
| beta-Hydroxymyristic acid | 306.755 | 243.1961113 | 0.971520462 | 2.022949065 | 0.017017822 | 0.102589342 |
| (2-oxo-2,3-dihydro-1H-indol-3-yl) acetic acid | 127.5425 | 190.054097 | 0.970186538 | 1.541078847 | 0.042547383 | 0.155384274 |
| AKLOMIDE | 25.61425 | 198.9915161 | 0.969978615 | 1.094213402 | 0.00221902 | 0.04353915 |
| Cucurbitacin I | 305.131 | 513.2883327 | 0.963455538 | 1.425106336 | 0.011400284 | 0.085952603 |
| Taurocholic acid | 257.0505 | 514.283484 | 0.957381231 | 1.902695368 | 0.023784223 | 0.119366022 |
| L--Hydroxyisovaleric acid | 35.98 | 117.055638 | 0.955693846 | 1.857421405 | 0.018710613 | 0.106637698 |
| 17-U-46619 | 419.851 | 347.2255988 | 0.954631692 | 1.4140137 | 0.018253217 | 0.105501115 |
| 6-Benzylaminopurine | 160.81 | 226.1070249 | 0.986471308 | 1.385821859 | 0.00637171 | 0.046069511 |
| Pentanoic acid | 103.7935 | 101.0607466 | 0.953348154 | 1.519275476 | 0.036866257 | 0.145649052 |

rt: Chromatographic retention time of the substance

mz: The mass-to-charge ratio of the characteristic ions of a substance

score: Qualitative matching scoring, taking values [0, 1], the larger the better

VIP: The importance of the variable projection obtained by the OPLS-DA model of the substance in the group of comparisons

P-VALUE: The P value obtained by the t-test of the substance in the comparison of the group, P value = the probability that the hypothesis is correct but rejected = the number of negative results / the total number of results, which is a test probability of the sample data

Q-VALUE: The hypothesis test statistic (P value) is the result after the multiple hypothesis test is corrected. Q value = probability of being rejected but correct = number of false positive results/number of presumed positive results, which is an inference from the statistical test A kind of test probability, re-statistics of P value
